# Pediatric Regional Anesthesia: A Practical Guideline for Daily Clinical Practice

**DOI:** 10.1097/ALN.0000000000005554

**Published:** 2025-06-17

**Authors:** Peter Marhofer, Markus Zadrazil, Philipp L. Opfermann

**Affiliations:** 1 Department of Anesthesia, Intensive Care Medicine and Pain Medicine, Medical University of Vienna, Vienna, Austria.; 2 Department of Anesthesia, Intensive Care Medicine and Pain Medicine, Medical University of Vienna, Vienna, Austria.; 3 Department of Anesthesia, Intensive Care Medicine and Pain Medicine, Medical University of Vienna, Vienna, Austria.

## Abstract

The past two decades have seen remarkable progress in pediatric regional anesthesia. Significant efforts have been made to develop central and peripheral techniques that are both practicable and reliable, with increasing success and very low complication rates driving a growing appreciation for this subspecialty. Regional anesthesia can be used to optimize perioperative pain control, to avoid mechanical ventilation, and to take advantage of favorable immunomodulatory and gastrointestinal side effects in children. Implementing a broad spectrum of these techniques will require specialized knowledge of anatomic structures, experience to select appropriate techniques for specific surgical procedures, and considerable hand skills to execute these techniques. This review has been written to summarize state-of-the-art information about all relevant aspects of pediatric regional anesthesia and to provide a practical approach to how regional anesthesia in children can be implemented in daily clinical practice.

## Abstract 2

This review summarizes state-of-the-art information about all relevant aspects of pediatric regional anesthesia and provides a practical approach to how regional anesthesia in children can be implemented in daily clinical practice.

Children undergoing surgery require particular attention during the entire perioperative period. Before the operation, an adequate concept of premedication should be implemented, aiming to reduce anxiety and to facilitate intravenous or inhalative induction of anesthesia. During the surgical procedure, tailored anesthesia concepts are obligatory to provide cardiorespiratory stability and optimal perioperative pain therapy. Particular attention is required because many anesthesia drugs are not approved for all age groups, and some pharmaceuticals may be associated with neurodegenerative effects.^1–3^ Opioids, in particular, should be used very conservatively to avoid short-term adverse effects such as respiratory depression^4,5^ and long-term adverse effects like reduced gastrointestinal mobility^6^ or immune depression.^7^ In the postoperative period, an imperative task is to provide appropriate pain therapy.

Regional anesthesia greatly contributes to minimizing the perioperative use of opioids with optimal effect on perioperative pain. No less important are its “bonus effects” of avoiding gut paralysis or postoperative respiratory support and its nonsurgical indications like treatment of limb ischemia. What is more, regional anesthesia became reliable and safe in all age groups with the introduction of ultrasound to daily clinical practice.^8,9^ The past two decades have seen significant developments that more or less redefined, through the use of ultrasound guidance, all useful techniques of regional anesthesia in pediatric patients of any age.^10^ Thus, when appropriate, regional anesthesia can be considered as the most important pillar in perioperative pain therapy.

Three elements need to be present for regional techniques to be fully implemented in pediatric anesthesia: structured education, continuous training, and a high case load. Once regional blocks can be performed at a high level of competence (*i.e.*, fast performance of procedures, high success rates, very low complication rates), it will also be possible to avoid side effects like cardiorespiratory impairment.^11^ For this reason, there is a need for educational concepts to be developed in pediatric anesthesia. Bodies like the Society of Pediatric Anesthesia (Richmond, Virginia) and the European Society of Pediatric Anesthesia (Prague, Czech Republic) are instrumental in providing state-of-the-art education *via* expert-based opinions and publications or specific workshops to a large community of anesthesiologists specializing in pediatric anesthesia.

Just as important, an integrated system needs to be established for pediatric regional anesthesia that encompasses all staff both in the operating room and on the wards.^12^ Anyone with a job in pediatric surgery may be assumed to believe that pain therapy should be optimized and side effects and complications minimized. Operating room staff is very grateful for, and motivated by, smooth perioperative workflows taking place in a calm and serene environment. Thus, the systematic use of pediatric regional anesthesia requires education and support not only from anesthesiologists but also from our partners in the operating theater and on the wards. The same is true of our fellow professionals who expect a focused and safe anesthesiological management during their surgical procedures.

This review article cannot replace a textbook. Its purpose is to create an overview of what, at the time of writing, is state of the art in pediatric regional anesthesia. The authors will discuss the most relevant and reliable techniques of regional anesthesia in children of different ages, presenting ingredients of action and suggesting ways to work these into recipes. Numerous centers around the globe have developed their own concepts of perioperative management in pediatric anesthesia. Against this background, the current article has been written to reflect the authors’ own clinical practice, based on three decades of experience with ultrasound-guided regional anesthesia that is strongly grounded in evidence-based medicine.

In other words, all the techniques discussed throughout this article have their roots in translational science. Care has been taken to follow a consistent anatomic rationale that can be readily understood. It is beyond the scope of this text to cover each and every technique of pediatric regional anesthesia. A strong focus lies on techniques with a clear anatomic background. In this light, the techniques of perioperative pain therapy that will be discussed do cover the entire spectrum of pediatric surgery to provide a practical approach for how regional anesthesia in children can be implemented in daily clinical practice.

## General or Regional Anesthesia, or Both?

Most regional blocks in pediatric anesthesia are still performed under general anesthesia. This is despite the fact that drugs routinely used for this purpose have been associated (though final confirmation of this suspicion has yet to be provided) with impaired neurodevelopment in children, especially during the neonatal age range.^13–15^ Recent studies also show neurodevelopmental implications of general anesthesia in older age groups (2 to 5 yr).^16^ Although all conclusions from these studies need to be interpreted with caution, it appears obvious that any reduction of general anesthetic and analgesic drugs may be beneficial, or formulated the other way around, less general anesthetic and analgesic drugs are unlikely to be harmful to our patients.

The implementation of ultrasound in the clinical practice of pediatric regional anesthesia with subsequent improved block qualities was the major boost to perform surgical procedures without involving opioids or invasive airway manipulation. Neuraxial and upper-limb blocks, due to their well-known sensory distribution patterns, were the prime candidates. Hence, this strategy has been pursued for arm/hand^17^ and inguinal^18^ surgeries, but even procedures of a more invasive kind, like pyloromyotomy^19^ or ureteric reimplantation,^20^ are manageable without invasive airway manipulation, as are laparoscopic procedures.^21^

Obviously, regional anesthesia in children cannot, as a general rule, be performed without any kind of sedation. Selecting the most appropriate sedation regimen and management depends on many factors, including age, sociocultural behavior of the child and their parents, infrastructure at the center, and appropriately qualified staff.

Different methods of perioperative sedation are used with regional anesthesia. In children over 2 yr old, we prefer oral premedication.^22^ The rectal route can be used in younger children and cases posing other challenges, whereas no premedication is needed in infants less than 6 months of age. Even though older children will allow sedation to be induced through a vascular access, we usually administer sevoflurane *via* a face mask with subsequent establishment of a vascular access. Emla cream, applied to the skin surface 45 min in advance, is useful to reduce the pain stimulus during puncture for regional anesthesia. Once the block has been performed, sedation is continued with propofol (5 to 10 mg · kg^−1^ · h^−1^) but can be significantly reduced after caudal blockade in babies, possibly due to loss of sensory input of the lower body.^23^ Research into nonpharmacologic sedation regimes, as a potential alternative to propofol, is ongoing.^24,25^

## Enhanced Recovery After Surgery and Pediatric Regional Anesthesia

One of the main advancements in surgery and anesthesia is Enhanced Recovery After Surgery (ERAS). In the field of anesthesia, ERAS is mainly based on postoperative pain therapy with the limitation of opioid use, the use of nonopioid adjuncts, and the implementation of regional anesthetic blocks.^26^ In particular, regional anesthesia is a controversial topic in this field, with an only moderate quality of evidence,^26^ but initial studies highlight the implementation of regional anesthesia in children as part of ERAS protocols.^27^ The use of ultrasound-guided regional anesthesia, a nowadays standard technique, in the concept of ERAS is only marginally described in adults.^28^ Nevertheless, all regional anesthetic techniques, which are described throughout this review article, show a potency to be included in future ERAS guidelines for children. The perioperative anesthetic management of pyloric stenosis *via* single-shot epidural anesthesia and sedation may serve as only one example in this context,^19,29^ but future studies are required to create more knowledge in that field.

## Identifying the Anatomic Structures

Locating the relevant anatomic structures is the single most important requirement for successful regional blocks. Historically, surface landmarks, in combination with the paresthesias they provoked, were the only way to approximate the targeted nerve structures. In other words, the paresthesia had to be detected before the local anesthetic could be administered.^30^ In children under deep sedation and general anesthesia, however, paresthesia cannot be detected, and what is more, direct needle–nerve contact is today known to cause nerve injury, which can be quantified as decline in compound muscle action potential.^31^

Beginning in the 1970s, nerve stimulation was introduced to the clinical practice of regional anesthesia,^32^ and it was not until the early 2000s that ultrasonography had evolved to the point of setting a new standard.^8^ After a decade of clinical experience in adult patients and greatly facilitated by the development of portable and handheld ultrasound equipment, the ground was prepared for using this technology also to identify nerve and adjacent anatomic structures in children. The initial literature illustrated the superiority of ultrasound in comparison with nerve stimulation regarding shorter onset times of block, longer duration of blocks, and improved sensory and motor block scores. In cases of trauma surgery (*e.g.*, fractures), the performance of blocks were less painful.^33,34^

Not only the downsizing but also the decreasing cost of appropriate equipment during that time promoted a more widespread use of ultrasound for regional blocks. Given the closer skin-to-nerve distances in children, it was, in effect, a natural consequence for these technological developments to redefine the range of applicable regional techniques for pediatric anesthesia. Notable examples of such applications would include ilioinguinal blockade,^35^ epidural catheterization,^36^ and brachial plexus blockade.^33^

Despite being widely used, ultrasound guidance for regional anesthesia continues to be somewhat controversial, although, in all fairness, it is not so much the scientific literature as personal communications that may create a perception of its usefulness being occasionally questioned. In fact, no proof of a decreased complication rate (*i.e.*, short-term or permanent neurologic deficit, inadvertent intravascular administration of local anesthetics) associated with ultrasound-guided regional blocks has ever been provided. While this may also be down to an incidence of complications so low (*e.g.*, 0 to 0.4:10,000 for permanent neurologic deficits^37^) as to preclude further analysis, the current body of evidence still supports direct visualization of anatomic structures by ultrasound as today’s gold standard for specific regional blocks in pediatric anesthesia.

## Pharmacologic Knowledge Requirements: Preventing and Managing Local Anesthetic Systemic Toxicity

Local anesthetics, in children, are associated with larger distribution volumes, longer half-lives, and less protein binding than in adults. Administration of local anesthetics to infants up 6 months old will result in a comparatively larger free proportion of the drug, as the main proteins to which these substances bind (α_1_-acid glycoprotein and albumin) are less abundant. Signs and symptoms of toxicity are invariably caused by the free and hence pharmacologically active fraction of any local anesthetic.^38^

To illustrate how significantly these pharmacokinetic differences vary with age, the relevant data for 0.75% ropivacaine are stratified by age and weight groups in table 1. As a rule of thumb, smaller children are more sensitive to local anesthetics, implying that lower doses will suffice for equieffective pharmacodynamics, and have to deal with larger free fractions of the drug, increasing the risks of toxicity at younger ages.

**Table 1.** Estimated Pharmacokinetics for Pediatric Age Groups from Pooled-population Pharmacokinetic Analyses

Characteristic	Newborns	1 Month	6 Months	1 yr	4 yr	10 yr
Median body weight, kg*	3.27	4.29	7.85	10.15	16.69	32.19
Clearance of unbound ropivacaine, l/kg	2.40	3.60	8.03	11.32	15.91	13.94
Ropivacaine distribution volume, l/kg†	21.86	25.94	41.71	52.60	65.24	65.57
Ropivacaine clearance, l · h^−1^ · kg^−1^	0.096	0.143	0.320	0.451	0.633	0.555
Ropivacaine terminal half-life, h	0.3	5.0	3.6	3.2	2.6	3.3

Source: Summary of product characteristics ropivacaine-HCl B. Braun (Germany) 7.5 mg/ml.

* According to the World Health Organization (Geneva, Switzerland) database.

† Also refers to the unbound fraction.

Recommendations for local anesthetics and adjuvants in pediatric anesthesia have been issued in a joint statement by the European Society of Regional Anaesthesia and Pain Therapy (Geneva, Switzerland) and the American Society of Regional Anesthesia and Pain Medicine (Pittsburgh, Pennsylvania).^39^ For epidural regional blocks, they recommend 2 mg/ml ropivacaine with a maximum dose of 2.5 mg/kg, and for peripheral nerve blocks, they recommend 0.5 to 1.5 mg/kg bupivacaine and ropivacaine, both based on low-level evidence (B2), along with dexmedetomidine as adjuvant (higher evidence level: A2). For epidural anesthesia, Bösenberg *et al.*^40^ investigated the pharmacokinetics of 0.2% ropivacaine applied continuously for up to 72 h at 0.2 ml · kg^−1^ · h^−1^ in infants aged 180 or less or at 0.4 ml · kg^−1^ · h^−1^ in infants aged greater than 180 days. Both regimens appear safe, considering that even higher concentrations of unbound ropivacaine in the younger group remained below the reported threshold of 0.35 mg/l for central nervous system toxicity in adults.^41^ Given these arguably very conservative dose recommendations for local anesthetics, we do use higher doses for epidural blocks in daily clinical practice (*e.g.*, for caudal blockade, 1 ml/kg 0.38% ropicacaine = 4.56 mg/kg^18^), but in accordance with the pharmacokinetic considerations just mentioned, take care to never exceed the lowest doses possible in very small children,^42^ regardless of which regional technique used. In addition, throughout the spectrum of regional blocks, our primary local anesthetic of choice is ropivacaine, which shows less systemic toxicity and causes less motor blockade when epidurally administered than bupivacaine.^43^ For extended block durations, if required, we use 1 to 2 µg/kg dexmedetomidine.^44,45^

Minimum doses for peripheral nerve blocks have been evaluated in volunteer studies, as for upper-limb nerves in adults, which yielded a 95% effective dose (ED_95_) of local anesthetics for 0.11 ml/mm^2^ of cross-sectional nerve area.^46^ For sciatic nerve blockade, a clinical study in surgery patients found that 0.15 ml/mm^2^ of cross-sectional nerve area was effective.^47^ Similar research on ilioinguinal–iliohypogastric nerve blockade in children revealed a concentration requirement for ropivacaine of 0.21%.^48^ Ten years earlier, in another up-and-down protocol, 0.075 ml of local anesthetic (0.25% levobupivacaine) had been found to be sufficient for ilioinguinal–iliohypogastric nerve blockade in a surgical setting.^49^ The exact peri-epineural administration of local anesthetic *via* ultrasound guidance and findings that a complete circumferential spread of local anesthetic is not required for successful blockade^50^ serve as fundamental basis for such low-volume peripheral nerve blocks.

It is important to always distinguish clearly between volumes and concentrations of local anesthetics for regional anesthesia. Trying to compensate for a low volume by increasing the concentration is not an option, and neither is the other way around.

A caudal access, to name but one example, requires a specific volume for an adequate sensory block to be achieved cranially, and it takes a specific concentration to achieve a defined intensity of sensory (and perhaps even motor) blockade.^51^ “Volume blocks” are usually needed for epidural anesthesia or fascial plane blocks, while “concentration blocks” are required to block peripheral nerves. Extra care should be exercised in performing fascial plane blocks, as precise administration between fasciae will result in faster absorption and higher plasma levels of local anesthetics,^52^ emphasizing once again the importance of striking a smart balance between volume and concentration.

“Local anesthetic systemic toxicity” occurs rarely in children, given a reported incidence of 8 in 100,000 regional anesthetic procedures.^53^ Manifestations include loss of consciousness (provided the patient is awake during the block) with or without tonic–clonic convulsions and various cardiovascular signs such as bradycardia, any tachyarrhythmias, and asystole. Most cases on record affected infants and have been associated with doses of local anesthetics not exceeding current recommendations.^53^ Cardiovascular collapse is adequately treated by resuscitation and intravenous administration of fat emulsion,^54,55^ with mainly favorable outcomes on record for lipid rescue therapy consisting in 20% Intralipid at 1.5 ml/kg greater than 1 min, continuous infusion at 0.25 mg · kg^–1^ · min^–1^, and repeated boluses twice in 5-min intervals.^53^

## Education and Training Strategies

Education is the most important enabler of regional anesthesia in children. Without a doubt, the introduction of ultrasound to daily clinical practice gave a boost to all types of regional blocks and has increasingly stirred anesthesiologists’ interest in pediatric regional anesthesia. Its availability did not, however, raise the quality of regional anesthesia all by itself, considering that it also takes specialized theoretical knowledge and hand skills for these techniques to be performed safely and effectively.

Topographic anatomy and pharmacology are two quintessential areas of knowledge underlying the practice of regional anesthesia, which, by one of its godfathers, Alan Winnie, was famously equated with “applied anatomy.” Most complications known to be associated with a specific technique can be safely avoided by practitioners who know about the relevant anatomic structures and their variants. The pharmacologic knowledge requirement, in turn, implies that practitioners need to acquire a detailed understanding of the activities exerted by local anesthetics and their adjuvants.

More of a challenge than to convey the requisite theoretical knowledge is to teach the hands-on skills for ultrasound-guided regional anesthesia, specifically regarding the mastery of needle guidance and hand–eye coordination. It is important to understand that some of the regional techniques here discussed are not based on ultrasound guidance alone. In practice, legacy principles of guidance such as “loss of resistance” are still important pillars of regional anesthesia, often utilized in conjunction with ultrasound guidance.

Little evidence-based literature is available to define teaching strategies for pediatric regional anesthesia. The three studies below do present findings consistent with our own clinical experience and are based on reliable data; hence, we believe that these findings give a realistic indication of the case loads required for anesthesia in children.

Schuepfer and Jöhr^56^ evaluated the learning curves for penile nerve blocks performed by residents on neonates, infants, and children. Failure rates decreased from 9% after 10 blocks to 4% after 20 blocks performed; after 40 and more blocks, the success rate had reached 94% with no significant additional gains. Ford *et al.*^57^ noticed a requirement of around 20 ultrasound scans for trainees to appropriately identify the muscle layers and nerve structures for ilioinguinal–iliohypogastric nerve blocks, and research by Moore at al.^58^ into the usefulness of simulating ultrasound guidance for regional anesthesia revealed a direct association between more training and better clinical performance.

On the other hand, not all regional block techniques are alike. Different nerve blocks are associated with different levels of complexity, and data available for one procedure cannot be automatically extrapolated to other procedures. We propose a classification of regional nerve blocks that differentiates between three levels of complexity:

- *Basic:* Terminal branches of brachial plexus (ulnar/median/radial) – forearm and arm, femoral, saphenous, ilioinguinal–iliohypogastric, rectus sheath, dorsal penile nerve
- *Intermediate*: Supraclavicular, infraclavicular, sciatic
- *Advanced*: Thoracic paravertebral, neuraxial

This classification, modified from Marhofer,^59^ implies that training concepts ought to teach *basic* blocks first, followed by *intermediate* and, last, *advanced* blocks. High-level hand and multitasking skills are notably required for techniques, such as epidural anesthesia, that involve more than just one recommended approach to identifying anatomic structures. Incorporating up-to-date technologies like “virtual reality” or “augmented reality” appears to make good sense in the training of regional anesthesia. Tools of this type, in addition to improving educational effectiveness, would allow trainees to hone their skills in a virtual environment before working on real patients.^60,61^

It is challenging to define education strategies, because every institution shows individual requirements regarding the education of theoretical and practical knowledge, dependent on geographical site, size of the individual institution, and the general approach to specific medical topics. Theoretical knowledge (*i.e.*, anatomy, pharmacology) can be acquired through the available literature, and the successful implementation of theoretical knowledge in daily clinical practice needs guided practice.

## Regional Anesthetic Techniques in Pediatric Patients

Any techniques of regional anesthesia documented in adults are, almost invariably, suitable for use in children as well. Ultrasound guidance, first reported in the literature around two decades ago, has greatly improved the effectiveness and reliability of these techniques. Today, pediatric regional anesthesia has an excellent safety profile, with reports on complications being anecdotal.^62–65^

This chapter discusses the most useful techniques, which, given their clear anatomic rationale, can be successfully used by competent anesthesiologists whenever they are indicated in daily clinical practice. Table 2 provides an overview about the most suitable regional anesthetic techniques in pediatric anesthesia.

**Table 2.** Ultrasound-guided Regional Techniques

Block	Ultrasound Anatomy	Position of Probe	Needle Guidance (and Remarks)		Local Anesthetic	Additives
Caudal	Dura mater, conus medullaris	Paramedian longitudinal	Needle not visible	Observe spread of LA (anterior movement of dura mater)	Ropivacaine 0.375% (1–1.3 ml/kg)	Clonidine (1–2 µg/kg), dexmedetomidine (1–2 µg/kg)
Epidural	Dura mater, spinal cord	Paramedian longitudinal	Needle not visible	Observe spread of LA (anterior movement of dura mater, catheters visible)	Ropivacaine: Bolus: 0.375% (0.4–0.5 ml/kg) Continuous: 0.2% (0.2–0.4 ml · kg^−1^ · h^−1^)	Fentanyl (1 µg/kg), clonidine (0.6 µg/kg)
Paravertebral	Parietal pleura, vertebral body, superior costotransverse ligament (internal intercostal membrane)	Transverse	Out of plane (anterior movement of pleura)		Ropivacaine 0.475–0.75% (0.2 ml/kg) for four intervertebral levels	
Spinal anesthesia					Bupivacaine 0.5% (0.2 ml/kg)	Clonidine (1 µg/kg)
Supraclavicular	Prevertebral fascia, subclavian artery, pleura	Above clavicle, transverse	In plane (lateral to medial)		Ropivacaine 0.475–0.75% (0.5 ml/kg)	Dexmedetomidine (1–2 µg/kg)
Infraclavicular	Pectoralis major and minor muscles, clavipectoral fascia, axillary artery, pleura	Below clavicle, transverse to slightly oblique	Out of plane		Ropivacaine 0.475–0.75% (0.5 ml/kg)	Dexmedetomidine (1–2 µg/kg)
Median nerve	Superficial and profound flexor digitorum muscles	Mid-forearm, transverse	Out of plane or in plane		Ropivacaine 0.475–0.75% (0.1–0.2 ml/kg)	
Ulnar nerve	Flexor carpi ulnaris, superficial (humeroulnar head) and profound flexor digitorum muscles, ulnar artery	Mid-forearm, transverse	Out of plane or in plane		Ropivacaine 0.475–0.75% (0.1–0.2 ml/kg)	
Radial nerve	Brachial muscle	Above elbow, transverse	Out of plane or in plane		Ropivacaine 0.475–0.75% (0.1–0.2 ml/kg)	
Femoral nerve	Iliopectoral arch, femoral artery	Slightly distal to inguinal ligament, transverse	Out of plane or in plane		Ropivacaine 0.475% (0.1–0.15 ml/kg)	Dexmedetomidine 1 µg/kg
Saphenous nerve	Femoral artery, sartorius muscle	Lower third of thigh, transverse	In plane		Ropivacaine 0.75% (0.1–0.15 ml/kg)	Dexmedetomidine (1 µg/kg)
Sciatic nerve	Long head biceps and semitendinosus muscles	Mid-femoral, transverse	In plane		Ropivacaine 0.475% (0.1–0.15 ml/kg)	Dexmedetomidine (1 µg/kg)
Ilioinguinal– iliohypogastric	Transverse abdominis and internal oblique abdominis muscles, anterior superior iliac spine	Medial to the anterior superior iliac spine, transverse	Out of plane or in plane		Ropivacaine 0.75% (0.2 ml/kg)	Dexmedetomidine (1 µg/kg)
Rectus sheath	Abdominis rectus muscle posterior sheath, parietal peritoneum	Both sides lateral to umbilicus, transverse	Out of plane or in plane		Ropivacaine 0.75% (0.1 ml/kg on each side)	
Dorsal penile nerve	Cavernous body, deep penile (Buck’s) fascia	Between penis and scrotum	In plane		Bupivacaine 0.5% (0.1 ml/kg)	

Note: For pediatric applications, use high-frequency (12 to 15 MHz) linear probes only.

LA, local anesthetic.

## Neuraxial Techniques

### Caudal Blockade

The most popular technique of regional anesthesia in children is the caudal approach through the saccrococcygeal membrane to inject a local anesthetic, with or without an additive, into the epidural space. Caudal blockade is rarely used in adults because the sacrococcygeal membrane undergoes ossification during adolescence. In children, this technique is popular for being technically straightforward, for covering a wide range of indications, and for its high success rate and excellent safety profile.

Technically, the procedure of pediatric caudal blockade has significantly evolved over the years. The first report, published almost a century ago,^66^ introduced an approach based strictly on anatomic landmarks, with the sacral cornuae forming the sacral hiatus and the sacrococcygeal membrane as the caudal boundary of the epidural space. Usually, these structures can be readily palpated. With the child in a lateral position with knees flexed, a short beveled needle with an injection line can be used for puncture (whose success is indicated by a tangible loss of resistance), after which we recommend disconnecting the syringe to rule out a subarachnoid needle position.

**Fig. 1.**
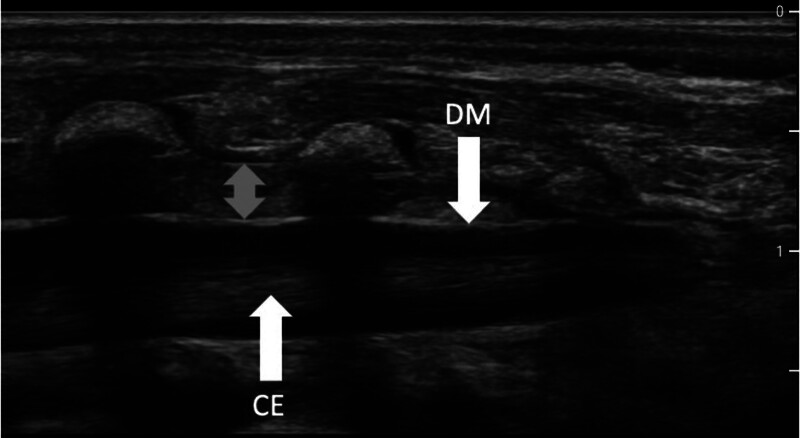
Longitudinal ultrasound image of the relevant anatomy for caudal blockade. The local anesthetic, which is administered during caudal blockade, is mainly visible by downward movement of the dosal part of the dura mater (DM). The *right side* is the caudal direction. (See also supplemental video 1, https://links.lww.com/ALN/E5.) CE, caudal equina.

Now, ultrasound imaging is used to endorse the strictly landmark-based approach,^67,68^ in which the dura mater, as visualized from a paramedian longitudinal position of the ultrasound probe, has become the key reference structure (fig. 1). The spread of injected fluid is clearly indicated by the dura moving in an anterior (or, from the perspective of imaging, downward) direction (supplemental video 1, https://links.lww.com/ALN/E5), and any inadvertent subcutaneous injection of local anesthetic is also readily detected in this way. In this event, the administration of local anesthetic should not be followed through before the dura mater is seen to move as required.

While the success rates achieved with the strictly landmark-based technique (success meaning that no systemic pain therapy was additionally required to perform a surgical procedure) did not exceed 80 to 90%, the introduction of ultrasound guidance raised this figure to almost 95 to 98%.^18,69^ Essentially, a caudal block will only fail if the sacrococcygeal membrane cannot be punctured due to extreme obesity or anatomic abnormalities.

Its main complication is inadvertent intravascular administration of local anesthetic. Kim *et al*.^70^ reported a 10.9% incidence of partially intravascular injections during caudal blocks, regardless of needle type (Touhy *vs.* Quincke) but directly associated with bone contact. In other words, the main cause of intravascular administration does not seem to lie in direct intravascular positioning of the needle tip but in instant absorption of local anesthetic by the vascular system after contact with bone.

As the second potential complication of caudal blockade, the eventuality of total spinal anesthesia should be taken into account,^71,72^ although the reported incidence is very low at 1:11,000.^73^ Careful observation of liquor backflow is the first management strategy to detect subarachnoid injection. It is also mandatory to check, as a sign of total spinal anesthesia, for dilation of the pupils right after local anesthetic has been administered. In sedated children with no airway manipulation involved, apnea and bradycardia will be the main clinical indications.^72^ While all consequences of total spinal anesthesia are readily manageable, paying attention and pursuing a clear clinical strategy (mainly in the form of cardiorespiratory support) are essential to achieving favorable outcomes.

Various local anesthetics are suggested for caudal blockade.^74^ Our policy is to invariably use 0.375% ropivacaine (a 1:2 mixture of 0.75% and 0.2%) in volumes of 1 or 1.3 ml/kg at all ages. This uniform regimen streamlines the daily clinical routines and helps to avoid drug-related mistakes. Ropivacaine was first described as safe and effective for pediatric caudal blocks by Ivani *et al.*^75^ and involves less cardiotoxicity as well as motor blockade than bupivacaine while offering the same block durations.^76^ Given a small but unpredictable correlation for caudally administered local anesthetics between injected volumes and cranial spread,^77^ while the latter is not affected by the speed of injection,^78^ we follow the injection through at a good pace after verification by ultrasound.

A number of drugs are available that, used as an additive to local anesthetics, can increase the durations of nerve blockade. For caudal blocks, two α_2_-receptor agonists are eligible, clonidine being more widely used, while dexmedetomidine (the more selective agonist) is catching up in popularity.^79^ Added at the usual dose level of 1 to 2 µg/kg, either drug can extend analgesia to twice its normal duration. While clonidine has been associated with respiratory depression in (former) neonates,^80^ our in-house standard of monitoring newborns of up to and including 60 weeks of postgestational age greater than 24 h after surgery will allow these events (usually manifesting as prolonged episodes of apnea) to be detected and adequately managed. Sedation, mainly observed with dexmedetomidine at doses greater than 2 µg/kg,^79^ is a desirable side effect of α_2_-receptor agonists intraoperatively but can lead to protracted stays in the recovery room.

Lipophilic opioids also can be used as additives for pediatric caudal blockade, with fentanyl normally being added at a dose of 1 µg/kg. While offering a prolongation effect on postoperative analgesia similar to clonidine, side effects are more prevalent, including respiratory depression, vomiting, or bradycardia.^81,82^ Other additive drugs that have been described for caudal blockade in children, like preservative-free S(+)-ketamine^83,84^ or tramadol,^85^ have never gained a foothold in daily clinical practice.

Thanks to exact research, pediatric caudal blockade has evolved into a technique that covers a broad spectrum of clinical indications in a growing number of patients. Today, we can perform blocks of this type with a safe pharmacologic profile in pediatric patients of all ages, from extreme neonates up to patients weighing 50 kg.^86^

### Epidural Blockade

In 1988, Adrian Bösenberg^87^ reported on thoracic epidural anesthesia in small children *via* the caudal route. While not technically challenging, what clearly argued against this approach was bacterial colonization from the nearby anus. McNeely *et al*.^88^ found 20% of epidural catheters thus inserted to be colonized with bacteria. Realizing that a more proximal access to the epidural space was in order, it was again Bösenberg *et al.*^89^ who described epidural catheterization in 240 neonates and babies with a puncture level between L3/4 and T6/7 and a distance from skin to epidural space of 3 to 12 mm. In a research effort to improve on this technique, direct visualization by high-resolution ultrasound was added to the conventional loss-of-resistance technique.^90^ It was found that the dura mater, suggesting itself as the key reference structure for an ultrasound-aided technique of epidural puncture, could be optimally visualized from a paramedian position of the ultrasound probe at lumbar and thoracic levels. Clinical confirmation of these findings was obtained in neonates and babies with 0.53 to 4.0 kg of body weight.^91^

**Fig. 2.**
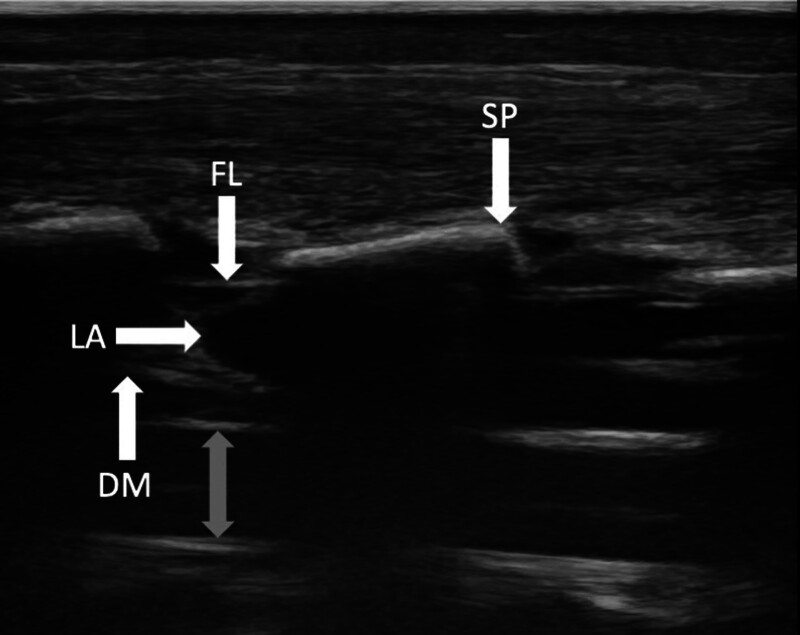
Longitudinal ultrasound image of the relevant anatomy for epidural blockade.The spread of local anesthetic (LA) appears from a caudal (*right side* of the image) to a cranial direction as hypoechoic and the epidural space distends between the flavum ligament (FL) and the dura mater (DM). The *gray* *double-headed arrow* indicates the spinal cord. SP, spinous process.

Note that, even in the form we use today, the method of epidural catheterization still relies on elements of both ultrasound and “loss of resistance” for guidance. From the paramedian longitudinal position of a high-resolution ultrasound probe, the path of a Tuohy cannula cannot be directly visualized. What can be visualized directly, adding significantly to the safety and reliability of this technique, is the correct spread of local anesthetic (fig. 2) and, in small children, the position of the inserted catheter. In addition, a significantly lower rate of bone contact is sustained for correct placement of the needle in the epidural space than with the loss-of-resistance technique used by itself (17% *vs.* 71%).^36^

Efforts aimed at optimizing epidural blocks in children have succeeded in expanding the spectrum of clinical indications. Today, even major and invasive procedures can be managed in this way, two examples being ureteric reimplantation^20^ and open surgery of pyloris stenosis^19,29^ performed under epidural anesthesia and sedation. The latter indication has always been somewhat controversial. Respiration tends to be impaired in children with pyloric stenosis, due to repeated vomiting and subsequent alkalinization of blood and cerebrospinal fluid, and given that their respiratory drive is often weak after surgery, a good case can be made for avoiding mechanical ventilation. Despite initial criticism,^92^ it appears that open surgery of pyloric stenosis can be managed (after careful aspiration of gastric content) safely and effectively under sedation by single-shot epidural administration of 0.75 ml/kg 0.475% ropivacaine at the thoraco-lumbar transition. This management exceeds the recommended dose levels but has proven to be safe in clinical practice.^19^

Laparoscopic procedures also can be managed under epidural anesthesia without airway manipulation. To name but one example, searching for nonpalpable testes by laparoscopic means would qualify for this approach.^21^ Close coordination with the surgeon is required for this strategy, whose success hinges on low intraabdominal pressures (less than 8 mmHg) and swift procedures to minimize the duration of laparoscopy.

Scoliosis surgery is another important indication for epidural catheterization. The catheter can be administered by the surgeon. Fentanyl (1 µg/kg) added to a low concentration of local anesthetic can be used for effective pain control and to avoid motor blockade.^93^

### Paravertebral Blockade

The paravertebral space, located bilaterally to the vertebral column, is bounded anteriorly by the parietal pleura and medially by the posterolateral aspect of the vertebral body, the intervertebral disc and foramen, and the superior costotransverse ligament (with its lateral continuation, *i.e.*, the internal intercostal membrane). The paravertebral space is not closed and has a direct communication to the prevertebral, intercostal, and epidural spaces. It contains the intercostal spinal nerves, the dorsal rami, the *rami communicantes*, the sympathetic chain, intercostal vessels, and fatty tissue.

Per-Arne Lönnqvist^94^ was the first author to describe paravertebral blockade in children for renal surgery and cholecystectomy and, in a follow-up study,^95^ reported on a failure rate of 6% and hypotension as the main complication. Karmakar *et al.*^96^ first described a bilateral paravertebral block, performed for thoracotomy in an 11-month-old infant.

Our own study group developed an ultrasound-guided technique for adults, finding that cross-sectional imaging was able to clearly identify the paravertebral space within the aforementioned boundaries.^97^ An magnetic resonance imaging study we performed next revealed a pattern of distribution with two-thirds of the local anesthetic spread in caudal and one-third in cranial directions, thus covering four segments.^98^ Lönnqvist and Hesser^99^ found a similar distribution of local anesthetics (four adjacent segments) with a volume of 0.5 ml/kg. Thompson and Haynes^100^ demonstrated the suitability of this technique for use in neonates, and Page and Taylor^101^ showed how paravertebral blockade was a good alternative to caudal and ilioinguinal blockade in abdominal surgery performed on children.

**Fig. 3.**
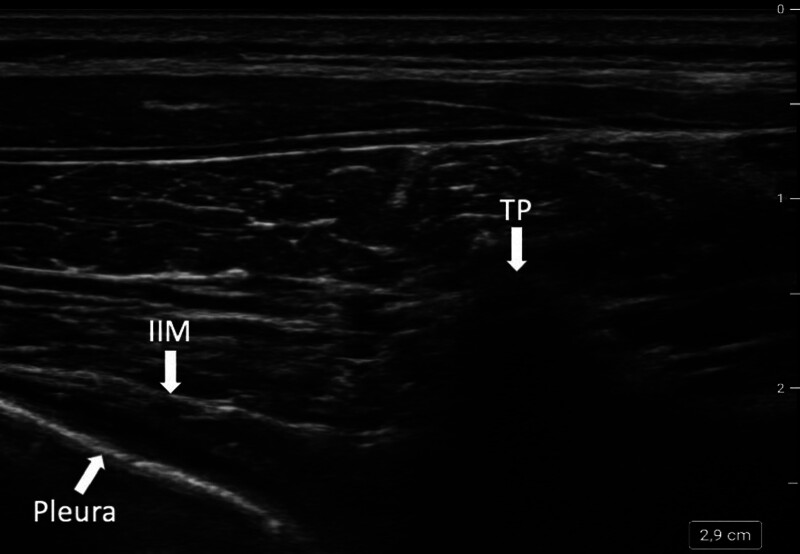
Cross-sectional ultrasound image of the paravertebral space, which is visible between the transverse process (TP), the pleura, and the internal intercostal membrane (IIM). The *right side* is the medial direction.

We recommend an ultrasound-guided approach with cross-sectional visualization of the paravertebral space to clearly identify its boundaries (fig. 3), combined with an out-of-plane or in-plane needle guidance technique. What dose levels should be used for paravertebral nerve blocks in children? Only rough approximations are given in the literature,^102^ but adult studies indicate that 20 ml of local anesthetic result in blockade of four intervertebral levels.^98^ Recalculating this dose for a child weighing 10 kg, 2 ml (0.2 ml/kg) would be required to block four intervertebral levels. That being said, for most surgical procedures, we recommend two punctures to block eight intervertebral levels.

### Spinal Anesthesia

Historically, the very first report on regional anesthesia in children, published in the early 1930s, dealt with a technique of spinal anesthesia.^103^ Two decades later, Berkowitz and Greene^104^ published a study on spinal anesthesia used in 350 children less than 13 yr of age.

The main asset of spinal anesthesia was that it avoided apnea in ex-preterm and small babies. According to a Cochrane review, the risk of postoperative apnea was reduced by about 50% in preterm infants subjected to inguinal hernia repair at a postmature age.^105^ The main liability of spinal anesthesia is its relatively short-lived effect in babies, reportedly not exceeding 60 min after administration of 0.5% bupivacaine (1 mg/kg = 0.2 ml/kg). Addition of 1 µg/kg clonidine can increase the block duration to twice as long without having any significant cardiorespiratory implications.^106^ Longer block durations of spinal anesthesia are achieved in older children.^107^

Given a strong research focus on caudal blocks, spinal anesthesia is not used that much in clinical practice. In a preterm baby weighing 1 kg, it is technically more challenging to administer 0.2 ml to the subarachnoid space than to administer 1 ml to the epidural space *via* the caudal route. In addition, the short block durations offered by spinal anesthesia are liable to result in stressful work environments, which a caudal block would avoid.

### Peripheral Techniques

Peripheral nerve block techniques can be performed *via* single-shot administration of local anesthetics with or without additive drugs or by continuous administration of local anesthetics *via* catheters. As illustrated in table 2, we prefer in the context of peripheral nerve block techniques single-shot administration due to the high percentage of catheter dislocations. Relevant literature regarding catheter dislocation rates is available only for the adult population.^108^ Specially designed catheters (*e.g.*, self-coiling catheters)^109^ and tunneling of the catheters^110,111^ may decrease the percentage of dislocation, whereas the puncture technique itself (in-plane *vs.* out-of-plane) does not influence catheter dislocations.^112^ It seems to be obvious that techniques in which catheters are introduced deeper under the skin are associated with dislocation rates. Thus, small infants and children are not really suitable for peripheral nerve block catheters. The hypermobility of awake children appears may also increase dislocation rates.

### Upper-limb Blockade

Since the brachial plexus can be anatomically accessed in various ways, a number of different approaches have been reported for use in both adults and children: distal to proximal, interscalene, supraclavicular, infraclavicular, and axillary. Dalens *et al.*^113^ were the first authors to elaborate, back in 1960, on brachial plexus blockade in children. They presented a strictly landmark-based axillary method with palpation of the axillary artery. Four injections were used: one subcutaneously over the artery, one above the artery, one posterior to the artery, and another one below and behind the artery.

What this description clearly indicates, and has since been anatomically confirmed, is that brachial plexus blockade *via* the axillary route is more of a peripheral nerve block. A different nerve may be blocked by each injection, such as the median nerve by the injection above the artery or the musculocutaneous nerve between the short head of the biceps and the coracobrachialis muscles once the needle has been advanced deeper. By today’s standards, this procedure may seem complicated, given four punctures and various manipulations of the cannula and the patient’s arm. The message, however, was ahead of its time, with the authors suggesting that regional nerve blockade should be established “as soon as the child reaches the hospital.”

In line with this “fast-track concept” by Dalens *et al.*,^113^ our policy is to perform brachial plexus blockade for trauma surgery immediately upon admission to the emergency room.^17^ Anatomic considerations have led us to favor periclavicular approaches. We can thus reach the entire brachial plexus through a single puncture to cover all surgical procedures at and distal to the level of the middle upper arm.^114^

De José María *et al*.^115^ reported a success rate of 95%, in the absence of any complications, with an ultrasound-guided technique for brachial plexus blockade *via* the supraclavicular approach. This is the easiest way to visualize the brachial plexus in its entirety lateral to the subclavian artery, using a high-frequency ultrasound probe and, preferably, in-plane needle guidance. For a safe block procedure, the pleura should also be clearly visualized. Particular attention is required when a dorsal scapular artery^116^ pierces the brachial plexus. In very small children, visualization of the brachial plexus may be impaired if the anatomic structures are too superficial to be clearly identified.

**Fig. 4.**
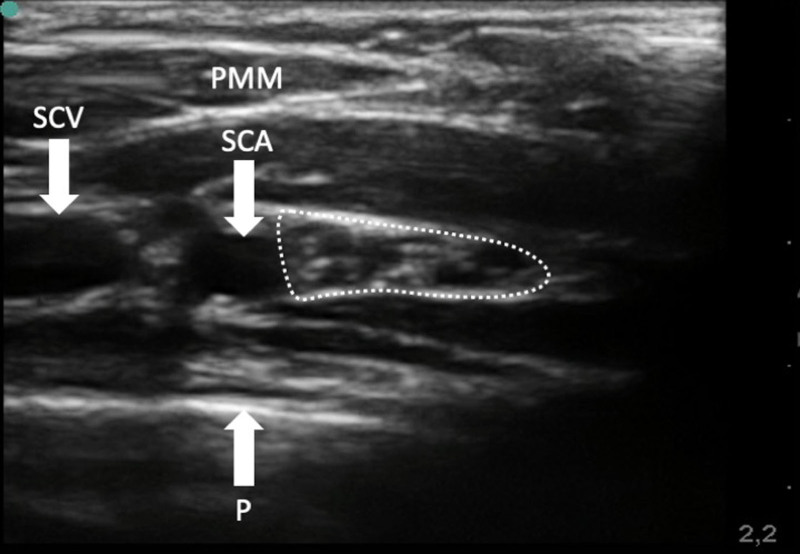
Cross-sectional ultrasound image of the infraclavicular portion of the brachial plexus (encircled by the *dotted line*) below the pectoralis major muscle (PMM) and lateral to the subclavian vein (SCV) and artery (SCA).The *right side* is the lateral direction. P, pleura.

Alternatively, the infraclavicular approach can be used, allowing all fascicles lateral to the axillary artery, as well as the pleura, to be visualized with the ultrasound probe in an oblique position perpendicular to the brachial plexus (fig. 4). Out-of-plane needle guidance should be used for the block procedure. Upon piercing the clavipectoral fascia, a single injection of local anesthetic is administered. Compared with nerve stimulation for guidance, this ultrasound technique cuts down on sensory onset times and offers longer durations of analgesia.^33^ Also, it should be noted that nerve stimulation close to an injury will be painful, whereas the ultrasound technique will not cause pain (not in the hands of an experienced operator).^33^ Infraclavicular blockade of the brachial plexus is considered safe for pediatric use^117^ and a feasible option even in very small children.^118^

Peripheral nerve blocks are appropriate for hand and finger surgery.^119,120^ Specific nerves that can be blocked for these indications conclude the median nerve, radial nerve, and ulnar nerve. It is worth noting that these nerves are characterized by highly variable patterns of sensory distribution; for details, the reader is referred to a volunteer study by Keplinger *et al.*^121^ On the other hand, all of them can be readily identified by ultrasound, and the blocks can be performed with very low volumes of local anesthetic.^122,123^ We recommend blocking the median nerve at the mid-forearm between the superficial and profound flexor digitorum muscles. The ulnar nerve can be blocked at the proximal third of the forearm between the flexor carpi ulnaris, superficial flexor digitorum (humeroulnar head), and profound flexor digitorum muscles. The radial nerve can be easily spotted above the elbow and below the brachial muscle.

### Lower-limb Blockade

Peripheral nerve blocks of the lower limb are not too well documented, presumably because all lower-limb surgeries can be managed by caudal blockade. This does not, however, change the fact that blocking the femoral and/or sciatic nerve can be useful whenever longer durations of postoperative pain therapy are required, as may be the case for indications of orthopedic surgery. Femoral and sciatic nerve blockade under ultrasound guidance is covered in the literature.^34^ To account for anatomic variations and their implications with regard to sensory distribution patterns and innervation of bony structures, it is common practice to block both nerves simultaneously.

The femoral nerve is visualized using a high-frequency linear ultrasound probe in the transverse plane lateral to the femoral artery and below the ileopectoral arch. In-plane (lateral to medial) and out-of-plane guidance of the needle are both options to administer 0.1 to 0.15 ml of local anesthetic.^124^ If motor blockade needs to be specifically avoided, a more distal approach to the femoral nerve can be taken for a saphenous nerve block.^125^ This branch of the femoral nerve is readily accessible from a position distal and medial to the thigh, close to the femoral artery, and below the sartorius muscle.

**Fig. 5.**
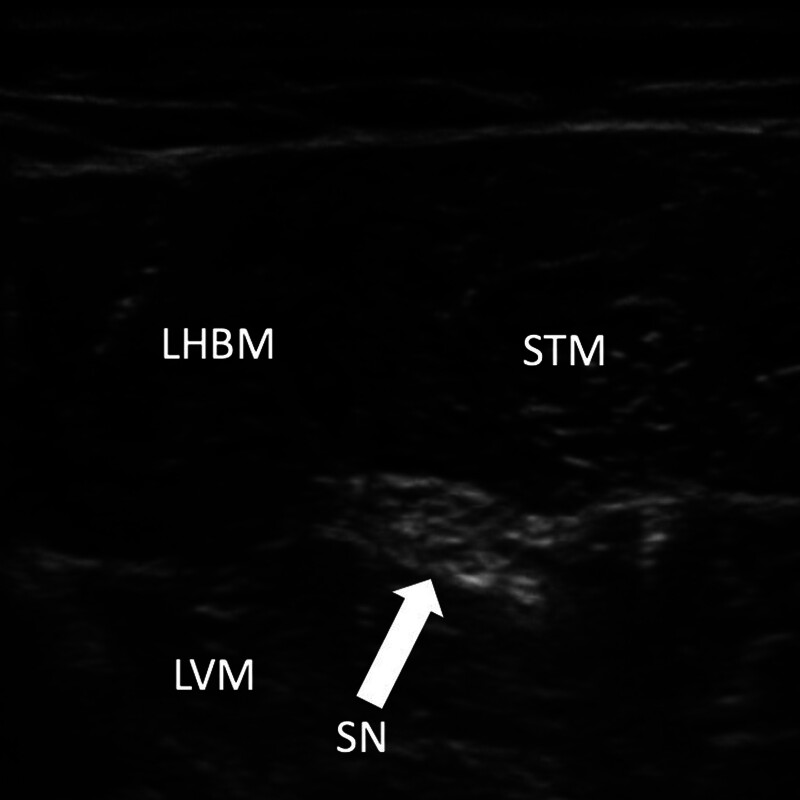
Cross-sectional ultrasound image of the sciatic nerve (SN) below the long head of the biceps (LHBM) and the semitendinosus (STM) muscles. The puncture is recommended *via* an in-plane needle guidance technique from lateral between the LHBM and the lateral vastus (LVM) muscles. This image also serves as example for the ultrasound appearance of peripheral nerve structures. The *right side* is the medial direction.

For the sciatic nerve, which can be imaged from gluteal to popliteal areas, we prefer a midfemoral approach with in-plane needle guidance from a lateral direction between the long head of the biceps and lateral vastus muscles (fig. 5). Its anisotropic behavior in ultrasound places higher demands on careful probe handling for the sciatic nerve to be optimally visualized. The same dose recommendation of 0.1 to 0.15 ml applies as for femoral nerve blockade. Selective blockade of the tibial or peronaeus nerve can be achieved by taking an approach distal to the bifurcation.

Long-acting local anesthetics will result in femoral and sciatic nerve blocks lasting for 8 to 12 h^34^ or indeed, with 2 µg of dexmedetomidine as additive, for up to 24 h.

### Truncal Blocks

Truncal blocks are fascial plane blocks in which the local anesthetic is injected between fascial layers, often without direct vision of the target nerves. We have previously expressed our skepticism about a relatively “novel” generation of fascial plane blocks, notable examples of which include the “erector spinae plane block”^126^ or the “serratus anterior plane block,” our main concern being their lack of a proper anatomic rationale.^127^ Hence, we shall confine our discussion of truncal blocks in this subsection to techniques supported by an adequate anatomic rationale and clear mechanism of blockade.

#### Ilioinguinal–iliohypogastric Nerve Block

Arising from the lumbar plexus and descending between the transverse abdominis and internal oblique abdominis muscles, the ilioinguinal–iliohypogastric nerves should always be blocked together; even the tiniest volumes of local anesthetic do not seem to block either nerve by itself.^128^ Various studies have revealed an effectiveness of blocking both nerves equal to neuraxial techniques for inguinal surgery.^129,130^

The initial method relied on “loss of resistance” slightly medial to the superior anterior iliac spine for guidance.^131^ A clinical study, including the use of ultrasound to verify this landmark-based approach, revealed that correct placement of the needle adjacent to the nerve structures was achieved in only 14% of cases, with a failure rate of 45%.^132^

Willschke *et al.*^35^ actually used ultrasound guidance for ilioinguinal–iliohypogastric nerve blockade in comparison with the landmark-based technique for inguinal surgery, showing that in-plane or out-of-plane needle guidance was possible and that surgical-grade anesthesia was attainable with 0.19 ml/kg of local anesthetic, compared to a volume requirement of 0.3 ml/kg for the conventional technique. The success rate was 96% for the smaller volumes with ultrasound guidance and 74% for the larger volumes with the landmark-based conventional technique.^35^ Subsequently, a dose-reduction study was performed and yielded a volume requirement of 0.075 ml/kg for inguinal surgery in children.^49^

#### Rectus Sheath Block

**Fig. 6.**
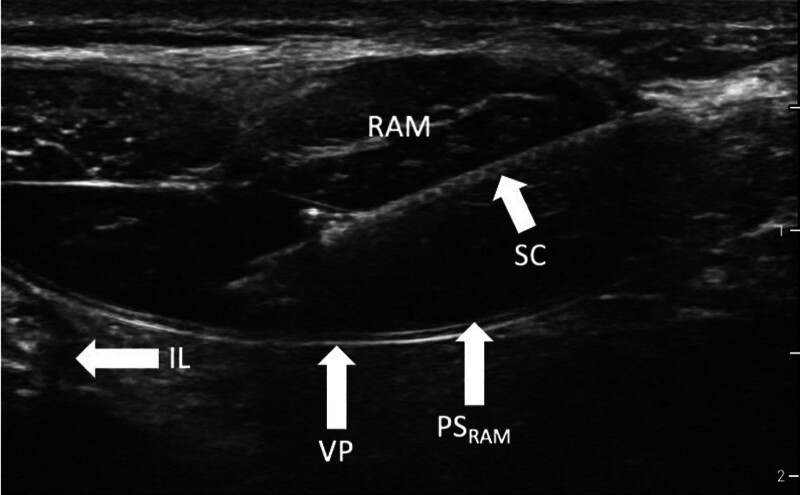
Ultrasound image of the rectus sheath block (one side), where the local anesthetic is administered below the rectus abdominis muscle (RAM) in the posterior sheath of the muscle (PS_RAM_). Care should be taken to avoid puncture of the visceral peritoneum (VP), where intestinal loops (IL) can be adjacent. The *right side* is the lateral direction. SC, shaft of the cannula.

This technique is employed for paraumbilical surgery only. We recommend it even though there is no way to directly visualize the target structures (*i.e.*, the terminal branches of intercostal nerves 9 to 11). Invariably, these blocks are carried out on both sides of the umbilicus, targeting the posterior sheaths of the abdominal rectus muscles. The ultrasound-guided technique, as described by de Jose Maria *et al.*^133^ and Willschke *et al.*,^134^ is to administer 0.1 ml/kg of local anesthetic on each side, using in-plane (fig. 6) or out-of-plane needle guidance. Dingeman *et al*.^135^ found that an exact ultrasound-guided technique performed by anesthesiologists, in comparison with a surgical infiltration technique, involved less postoperative pain and reduced opioid requirements. Caution is required in performing the puncture, as intestinal loops may be present along the needle path adjacent to the peritoneum, but if due care is exercised, this should not be a problem, as any anatomic structures inside the abdominal cavum can be visualized by ultrasound (fig. 6) and inadvertent puncture of the gut thus safely avoided.^136^

#### Dorsal Penile Nerve Block

Being an especially popular technique of pediatric regional anesthesia, dorsal penile nerve blockade was first described by Dalens *et al*.,^137^ who used short bevel needles to administer local anesthetic (0.1 ml/kg 0.5% bupivacaine) into the subpubic space. While reporting a 100% success rate, the definition of success they used falls short of today’s standard to always strive for opioid-free anesthesia as the final goal.

An ultrasound-guided technique for dorsal penile nerve blockade, using ropivacaine as local anesthetic, was described by Faraoni *et al.*^138^ The historical record for this method involves one reported case in which temporary ischemia of the glans penis was observed, possibly due to ropivacaine having an inherently vasoconstrictive quality.^139^

Zadrazil *et al.*^140^ developed an ultrasound-guided technique in which the high-frequency linear probe is placed between the penis and the scrotum, using in-plane needle guidance to administer the local anesthetic (0.1 ml/kg 0.5% bupivacaine) below the deep penile (*i.e.*, Buck’s) fascia. This technique offers a 100% success rate and allows surgery to be performed under mild sedation without any use of opioids.

## Discussion

Regional anesthesia is an essential pillar of perioperative treatment in children, and the introduction of ultrasound has significantly increased the popularity of pediatric regional anesthesia. In skilled hands, regional blocks are capable of providing opioid-free anesthesia and pain management. In addition, they can reduce the need for postoperative respiratory support, and compared to general anesthesia alone, many of them offer an additional potential for better outcomes *via* sympatholytic and immunomodulatory effects. Regional techniques for pediatric use are well documented in the literature, but broader contextual knowledge of regional anesthesia (*e.g.*, on the subject of immunomodulation and cancer recurrence) can be derived only from existing literature on adult patients.^141^

Having come a long way, we are today in a position to manage a wide range of surgical procedures mainly by ultrasound-guided regional anesthesia. Upper-limb^17^ and subumbilical surgery^18^ are perfect examples, but other procedures like thoracic^142^ and abdominal surgery^19^ are also being increasingly handled predominantly by regional techniques, and the same can be said of laparoscopic procedures.^21,143^ To successfully implement these strategies in daily clinical practice, a high level of collaboration with our surgical partners is essential. Regular interdisciplinary meetings and training sessions are useful in this to facilitate improved teamwork in the operating room.

Over and above surgical indications, regional anesthesia can be used to good effect for pain therapy during intensive care, sympatholysis, or gastrointestinal stimulation. Another notable indication would be the treatment of limb ischemia in neonates.^118,144^

Then there is the important topic of sedation, which can be handled in different ways for perioperative management strategies that are mainly based on regional anesthesia. Our preferred approach is to induce sedation by sevoflurane through a face mask with the child spontaneously breathing, followed by establishing a vascular access and performing the ultrasound-guided regional anesthesia technique. Sedation can then be continued using propofol.^18,69^ Babies less than 3 months old require lower doses of sedation after neuraxial techniques, presumably due to the block-related loss of sensory input.^23^ An interesting alternative is suggested by preliminary experience with nonpharmacologic sedation regimens.^25^

While pediatric regional anesthesia, in the hands of well-trained specialists, can take perioperative pain therapy to near perfection, it is important to remember that we still have an obligation to monitor our patients for pain beyond the immediate postsurgical period. No level of “technical” effectiveness can compensate for our children’s pain when the nerve block begins to resolve without us noticing. Block durations can be increased by catheter techniques or the use of additives to local anesthetics. That said, peripheral nerve catheters, in particular, carry a risk of dislocation.^108^ Additives to long-acting local anesthetics can prolong the effect of single-shot techniques significantly,^79^ but adequate pain control is normally achievable with nonopioid systemic analgesics by 48 to 72 h after surgery, the recommended approach being to overlap the “outgoing” effect of nerve blockade with the “incoming” effect of the analgesics. Patient- and nurse-controlled analgesia are also possible techniques to increase the efficacy of postoperative pain therapy, but its use decreased during the last years due to improved surgical techniques and the implementation of regional blocks in daily clinical practice.^145^

Future directions in the field of pediatric regional anesthesia should be based on excellent science regarding concepts to improve the quality of invasive postoperative pain therapy and to increase the worldwide implementation of pediatric regional anesthesia in daily clinical practice. The latter can be achieved only with the development of sophisticated educational programs.

Judging from the evidence accumulated to this day, regional anesthesia in children should be based on visualization of the relevant anatomic structures by ultrasound. The techniques we have discussed in this article are safe and effective if—and only if—performed by anesthesiologists with specialized knowledge and hand skills.

### Acknowledgments

The authors thank Wilfried Preinfalk, Mag. Phil. (Vienna, Austria), for language editing.

### Research Support

Support was provided solely from institutional and/or departmental sources.

### Competing Interests

The authors declare no competing interests.

## Supplemental Digital Content

Supplemental Video 1. Ultrasound controlled caudal blockade, where the correct spread of local anesthetic from caudal (right side) to cranial is clearly visible, https://links.lww.com/ALN/E5

## Supplementary Material
